# Animal Scanner: Software for classifying humans, animals, and empty frames in camera trap images

**DOI:** 10.1002/ece3.4747

**Published:** 2019-02-10

**Authors:** Hayder Yousif, Jianhe Yuan, Roland Kays, Zhihai He

**Affiliations:** ^1^ Department of Electrical and Computer Engineering University of Missouri‐Columbia Columbia Missouri; ^2^ Department of Forestry and Environmental Resources North Carolina State University Raleigh North Carolina; ^3^ North Carolina Museum of Natural Sciences Raleigh North Carolina

**Keywords:** background subtraction, camera trap images, deep convolutional neural networks, human–animal detection, wildlife monitoring

## Abstract

Camera traps are a popular tool to sample animal populations because they are noninvasive, detect a variety of species, and can record many thousands of animal detections per deployment. Cameras are typically set to take bursts of multiple photographs for each detection and are deployed in arrays of dozens or hundreds of sites, often resulting in millions of photographs per study. The task of converting photographs to animal detection records from such large image collections is daunting, and made worse by situations that generate copious empty pictures from false triggers (e.g., camera malfunction or moving vegetation) or pictures of humans. We developed computer vision algorithms to detect and classify moving objects to aid the first step of camera trap image filtering—separating the animal detections from the empty frames and pictures of humans. Our new work couples foreground object segmentation through background subtraction with deep learning classification to provide a fast and accurate scheme for human–animal detection. We provide these programs as both Matlab GUI and command prompt developed with C++. The software reads folders of camera trap images and outputs images annotated with bounding boxes around moving objects and a text file summary of results. This software maintains high accuracy while reducing the execution time by 14 times. It takes about 6 seconds to process a sequence of ten frames (on a 2.6 GHZ CPU computer). For those cameras with excessive empty frames due to camera malfunction or blowing vegetation automatically removes 54% of the false‐triggers sequences without influencing the human/animal sequences. We achieve 99.58% on image‐level empty versus object classification of Serengeti dataset. We offer the first computer vision tool for processing camera trap images providing substantial time savings for processing large image datasets, thus improving our ability to monitor wildlife across large scales with camera traps.

## 1 INTRODUCTION

Motion‐sensitive wildlife cameras, commonly referred to as camera traps, are increasingly popular survey tool for animal populations because they are noninvasive and increasingly easy to use (Kays, 2016). Comparisons with other wildlife monitoring methods have shown camera traps to be the most effective and cost efficient approach for many species (Bowler, Tobler, Endress, Gilmore, & Anderson, 2016). Ambitious projects are increasing the scale at which cameras are used on the landscape, now rotating hundreds of sensors across thousands of sites (Steenweg et al., 2016), sometimes with the assistance of citizen scientists (McShea, Forrester, Costello, He, & Kays, 2016).

Given the large amounts of pictures recorded by each camera trap, and the increasing number of cameras used by each study, processing and managing camera trap images has become a major challenge. For example, one two‐year study resulting 2.6 million images from 98,189 detections (McShea et al., 2016) across six states in the eastern USA. In some cases, false triggers and pictures of people can outnumber animal pictures. For example, with 1/3 of their cameras set on hiking trails (Kays et al., 2016) recorded 30,975 detections of people and 53,372 detections of wildlife in the eastern USA. Habitats such as savannas or forest canopies are particularly likely to produce false triggers due to vegetation blowing in the wind, for example, 98% (68,968 events) of all camera triggers in the forest canopy were moving vegetation (Gregory, Carrasco Rueda, Deichmann, Kolowski, & Alonso, 2014).

Most recently, deep neural network approaches have shown outstanding performance in image classification and object detection. Deep Convolutional Neural Networks (DCNNs) model is one of the most popular deep learning models that are widely used. A simple DCNN model consists of convolution, pooling, and classification layers. The convolution layers act as local and translation invariant operators between the input image and set of filters. The pooling layers are a down sampling using either max or average pooling. DCNNs learn features hierarchy all the way from pixels to classifier where the training is supervised with stochastic gradient descent (Lee, Xie, Gallagher, Zhang, & Tu, 2014). In this paper, the output from the classification layer is three scores which refer to human, animal, and background classes.

Computer vision has the potential to offer an automated tool for processing camera trap images if it can, first to identify the moving animal within the image and subtract the background, and second, to identify the moving object (He et al., 2016). Although these problems are solved for many indoor environments (Huang, Hsieh, & Yeh, 2015), the challenge is much greater with camera traps images because of their dynamic background scenes with waving trees, moving shadows, and sun spots.

Previous efforts to distinguish animals from background in camera traps have been proposed for foreground detection. In general, foreground areas are selected through one of two means, pixel‐by‐pixel, in which an independent decision is made for each pixel, and region‐based, in which a decision is made on an entire group of spatially close pixels (Dong, Wang, Xia, Liang, & Feng, 2016). Analytical approaches include constructing a background model using the median pixel value (Miguel, Beery, Flores, Klemesrud, & Bayrakcismith, 2016), a non‐parametric approach where the pixel‐level background model is represented by a set of background samples (Barnich & Van Droogenbroeck, 2011) and robust principle component analysis (RPCA) (Candès, Li, Ma, & Wright, 2011). Unfortunately, the success of these efforts has been limited by producing large number of false positives and difficulty in distinguish between animal and human objects.

## 2 METHODS

**Figure 1:**
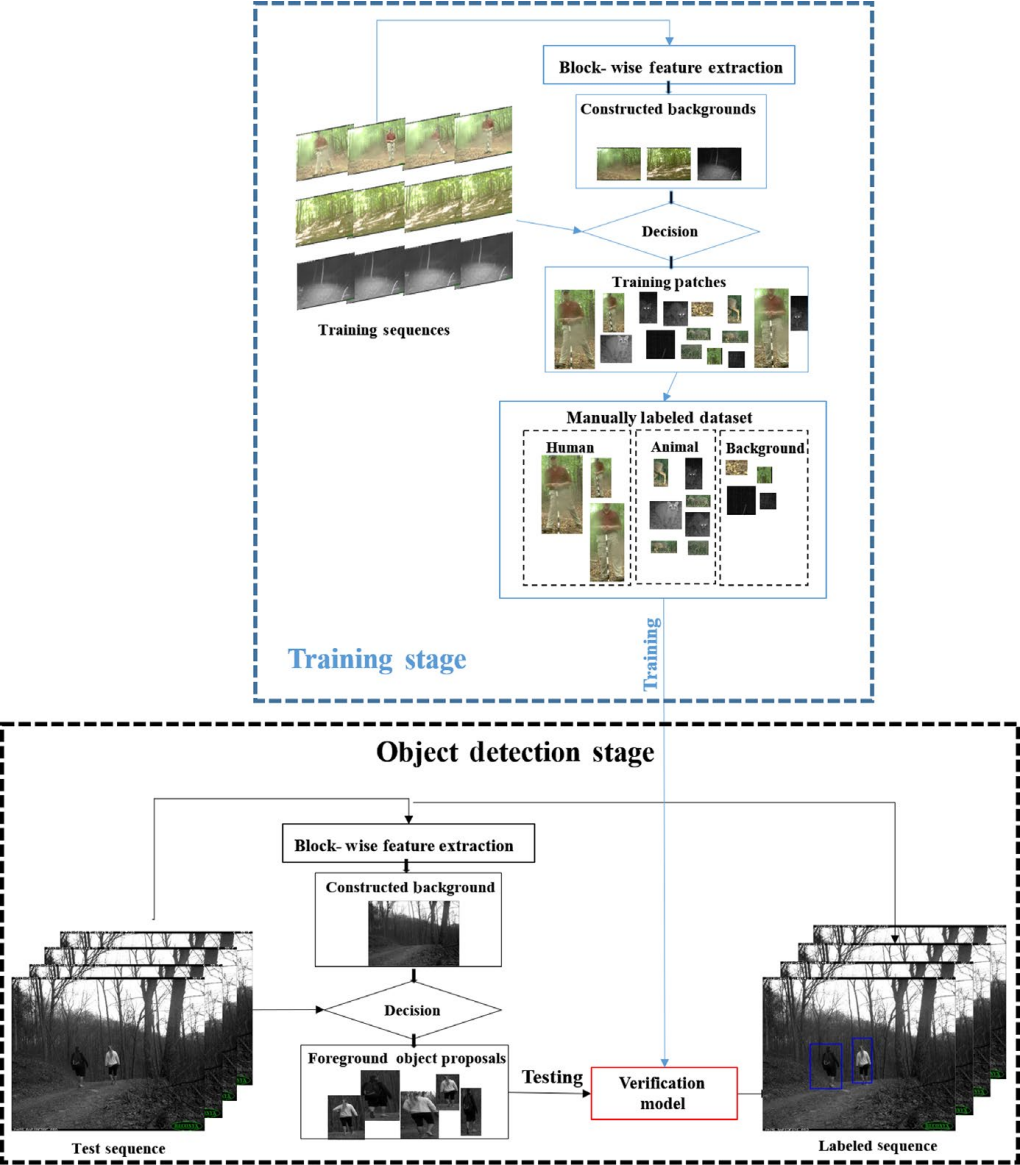
Flow chart of the proposed system. In the training stage, we generate the training patches to train the classification model. In the detection stage, we use joint background modeling with the pre‐trained classification model from the training stage to detect the human and animal

Our system (Figure 1) starts by detecting where the moving objects (human, animal, or moving vegetation) are within the images using a background subtraction method. Unlike many other image processing and vision analysis tasks, detecting and segmenting human–animals from the camera trap images is very challenging since natural scenes in the wild are often highly cluttered due to heavy vegetation and highly dynamic due to waving trees, moving shadows, and sun spots. Next, these moving objects are identified with classifiers to distinguish them as human, animal, or moving background. After describing these algorithms, we will explain how we reduce the false positives (background patches mistakenly identified as animals or people) using cross‐frame verification and present a study of the complexity‐accuracy trade‐off of DCNNs to propose a fast and accurate scheme for human–animal classification. Finally, we describe our GUI and command line data input and output.

### 2.1 Object region segmentation

The first step in processing camera trap images is to distinguish the moving objects in the foreground (aka foreground object proposals) from the fixed background. We rescale each frame from a given camera trap sequence into a specific width and height and then divide the rescaled image into 736 (or 32 × 23) regular blocks. We then extract features from each block. To determine which block(s) contain moving animals, we use the minimum feature distance (MFD) to all other co‐located blocks. These features include intensity, Local Binary Pattern (LBP) (Ojala, Pietikäinen, & Mäenpää, 2001), Gray Level Co‐occurrence Matrix (Baraldi & Parmiggiani, 1995), and Histogram of Oriented Gradient (HOG) (Dalal & Triggs, 2005). Given a sequence with 10 frames, for each block of the 736 blocks is compared with the other nine co‐located blocks to find the background block which has MFD. Any block that has feature histogram difference with the co‐located blocks larger than the MFD is classified as a moving object (i.e., foreground). Our experiments found that the HOG (Dalal & Triggs, 2005) is the best feature vector that can efficiently represent the block information.

We compare consecutive images in a sequence to find the moving object by subtracting feature histograms from same region position on subsequent images. The regions with the highest differences are then connected contiguously to form the moving objects. The difference value should be robust enough to reduce the number of false alarms and able to detect any animal or human as precisely as possible in the presence of challenges of camera trap images. Because some camera brands only record 3 images per trigger, we initially use information from three consecutive frames to find the moving object. In a second method, we use the entire sequence frames to extract a background frame in a composition manner, and then subtract each frame features histogram from this composite background. After we subtract a given frame from the background frame, we set a threshold value that defines whether this block belongs to background or foreground. The foreground blocks are then connected to represent the foreground region(s). These foreground regions are the region proposals which need to be verified as human, animal, or background in order to label them with tagged bounding boxes.

### 2.2 Region proposals verification

Before we proceed to the final step of identifying the moving object as an animal or person, we first use a verification of region proposals to determine if they are from the foreground or background. We observe that some of the false positive foreground generated by background subtraction are caused by the intensity changes within the same sequence.

**Figure 2:**
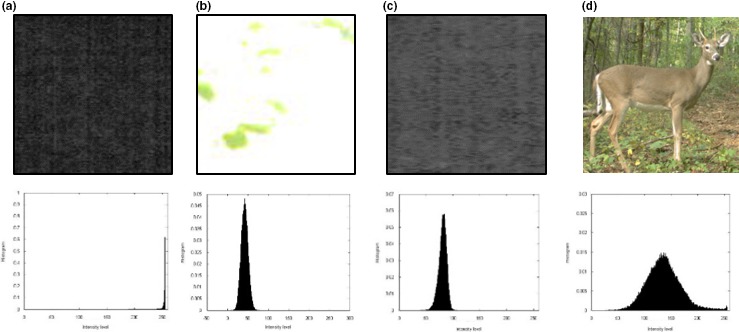
Foreground verification by histogram shrinking. The first row shows candidate foreground proposals identified by changing pixels between frames while the second row shows their intensity histograms. (a) Dark region far away from camera flash. (b) Sunspot region. (c) Gray region within the camera flash range. (a), (b), and (c) are false positive patches where the SHL values are very small, (d) is a patch where the SHL value is big

We define a threshold value called Shrinked Histogram Length (SHL) to determine whether an image patch is foreground (human or animal) or background based on the intensity information only. Let *n_i_* be the number of occurrences of intensity level *i*, then the SHL for an image patch of size *w* × *h* is:

$$\text{SHL}=\sum_{i=0}^{L-1}p_{i},\;p_{i}=\left\{\begin{array}{cc} 1 & \text{if}\;\frac{n_{i}}{\mathrm{wh}}>th_{\mathrm{hs}}\\ 0 & \text{otherwise} \end{array}\right.$$

where *L* is the total number of intensity levels in the patch. Figure 2 shows how the false alarms are detected through the SHL value. Region verification through SHL requires less than 50 ms for each patch.

### 2.3 Foreground proposals classification

After finding objects within a given frame (aka proposals), the next step is to classify them into human, animal, or background. We created a training dataset of images from these three classes (human/animal/background) by cropping rectangular regions (aka patches) from 459,427 camera trap images and manually labeling them. These images were all from Reconyx or Bushnell brand cameras, and included color and black/white pictures with mainly two image resolution 1,024 × 1,536 pixels and 1,920 × 2,048 pixels. The original images come from three countries (Panama, Netherlands, and USA), and thus represent a great variety of types of animals and people. We use this dataset to train and test three different classifiers: Bag of visual word (BOW) (Fei‐Fei & Perona, 2005), AlexNet (Krizhevsky, Sutskever, & Hinton, 2012), and our DCNN model (AlexNet‐96). The input image size for AlexNet and BOW is 256 × 256 pixels and 96 × 96 pixels for AlexNet‐96. Our software can accept any size of camera trap images (i.e., 1,024 × 1,536 and 1,920 × 2,048). It should be noted that cropped region proposals (image patches), which have different sizes and aspect ratios, need to be rescaled to match with the classifier required input size. The training set is completely separate from the testing data and both include randomly chosen sequences with different camera trap circumstances including color and black/white, trail road, grass, and top‐tree images. Each of the training and testing dataset contains 30,000 image patches consisting of 10,000 patches for each class.

We evaluate the performance of our human–animal detection method on 200 camera trap sequences each consisting of 10 images. We manually labeled all animals and persons with bounding boxes in these 2,000 images. To evaluate the detection performance, we compared the segmented output patch and the manually labeled patches with the intersection over the union (IoU). We consider our classifier as accurate (true positive = TP) if the patch has an IoU ≥ 0.5 and is classified correctly (person, animal). Any background classified as human or animal, or any IoU ≤ 0.5 are False Positives (FP), and False Negatives (FN) are human and animal patches that are classified as background allowing us to calculate performance metrics: (a) Recall TP/(TP + FN); and (b) Precision (TP)/(TP + FP).

### 2.4 Software

We implemented our algorithms in two forms, a graphical user interface and command line, to facilitate use by camera trappers and also to make the individual components available to other computer programmers who want to modify it or incorporate it into other software.

### 2.5 Graphic user interface

**Figure 3:**
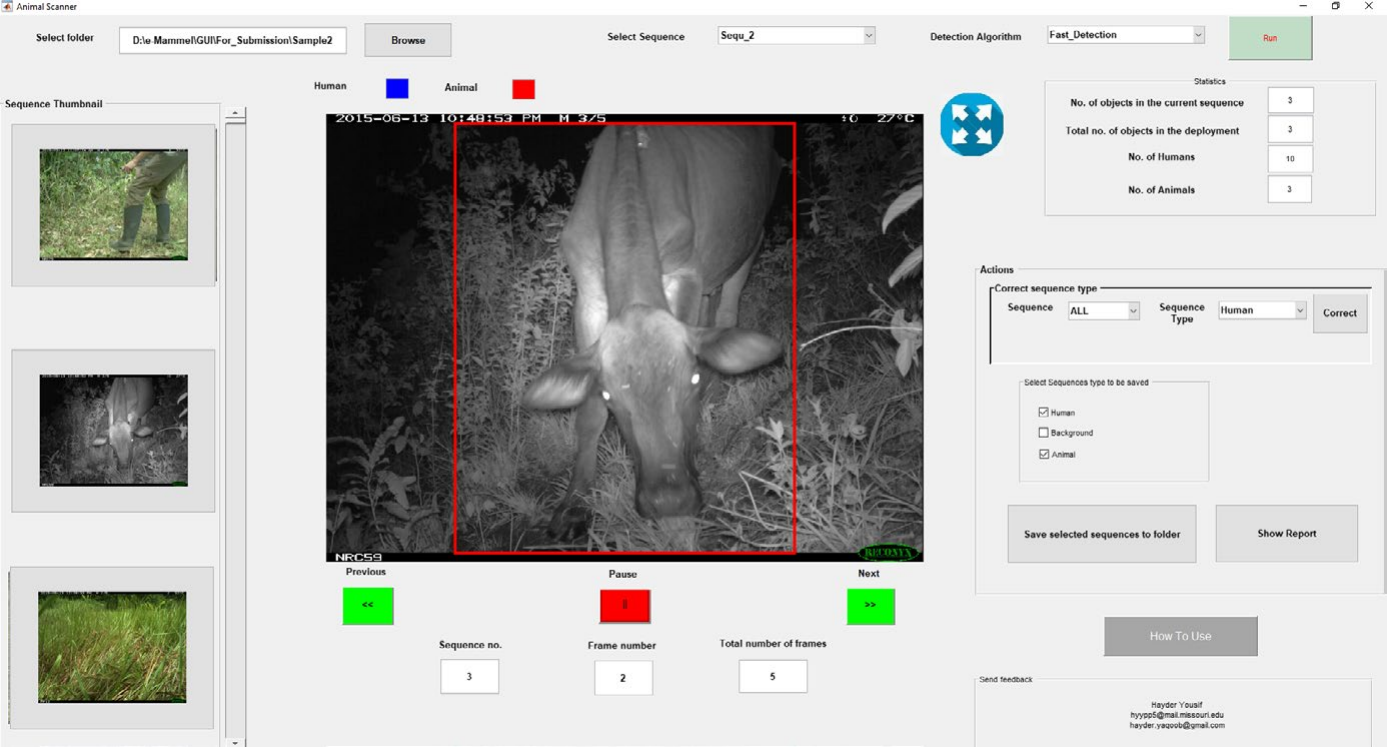
Screenshots from our designed GUI

**Figure 4:**
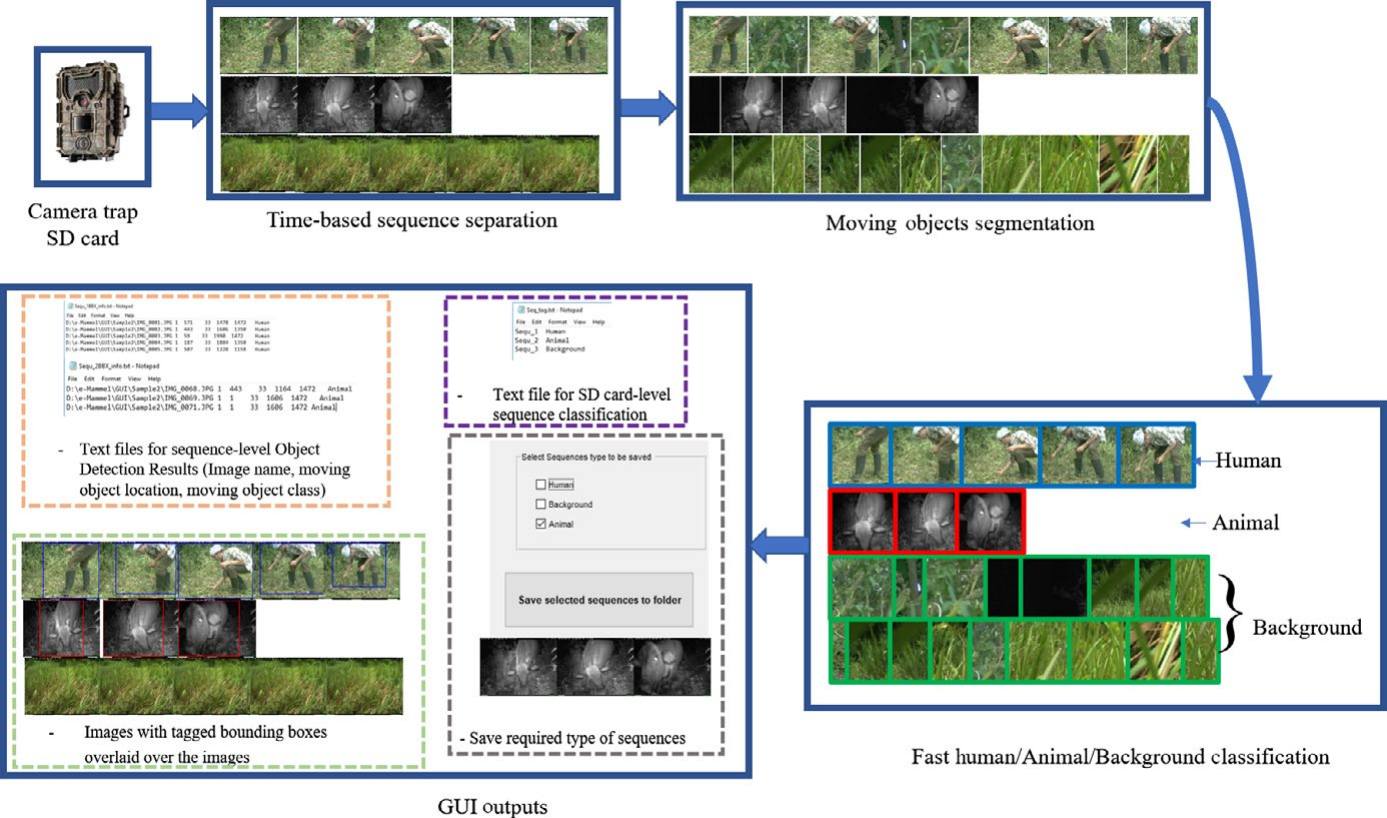
GUI image analysis flow

We have packaged our algorithms with a user friendly graphical user interface to allow ecologists to easily use our algorithms without detailed programing knowledge (Figure 3). The workflow (Figure 4) has the ecologist retrieving the *SD* card with images from the camera trap and uploading a series of images from one location (i.e., one camera trap deployment) into the software at once. The GUI automatically divides the deployment images into sequences based on time stamps by combining photos within 60 s of each other. The user can select one of the two background subtraction methods. Users can choose to run the software on the whole deployment or a specific sequence. The detection results (images with object bounding box) are stored as JPG images with the color of the box indicating the classification: blue for humans, red for animals, and no bounding boxes for background (e.g., blowing leaves or sunspots). The program shows statistics about the detected objects and saves the results for the full deployment as a text file. After processing, the user can choose delete human and/or empty sequences and save the filtered results into a specified folder. Figure 4 illustrates the main steps of our proposed software which includes: (a) sequence separation, (b) moving object segmentation using background subtraction, (c) region verification and fast DCNN classification, and (d) reports and processed images as output. The GUI is available in two versions: Windows^1^ and Linux^2^

### 2.6 Command line interface

We have developed a *C*/*C*++ command line interface program for fast human‐animal detection. The input argument required to run this program is only the program name and the input file that contains a list of all sequence images. Running the program on a batch of sequences requires a single text file contains the names of sequences files. For example, if I have 10 sequences, there will be 11 files, 10 files listing the name and the path of the images in each sequence, one file has the name and the path of each of the 10 files. Here is an example:

>>> *HumanAnimal*.*exe test*.*txt* 3

## 3 RESULTS

### 3.1 Fast DCNN analysis

**Figure 5:**
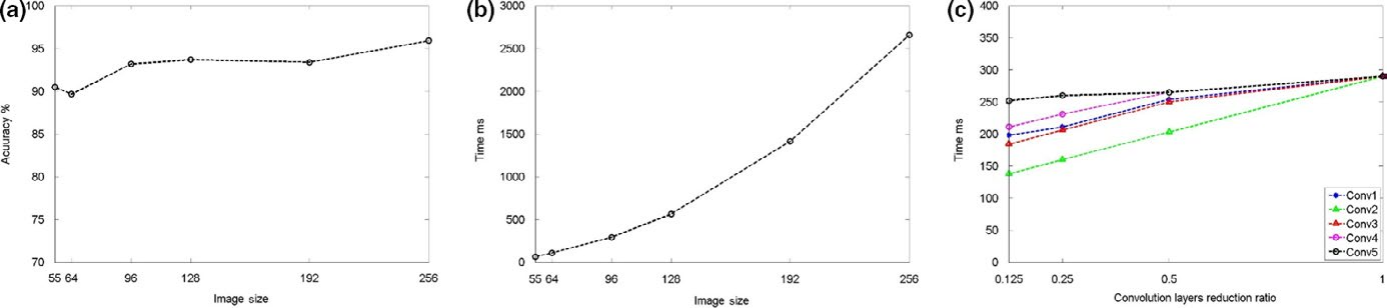
(a) Effect of input image size on the DCNN accuracy on a 2.6 GHz CPU and 16 GB RAM. (b) Effect of input image size on the DCNN execution time per patch. (c) Effect of reducing the number convolutional layer filters in each of the five convolutional layers (Conv) on execution time of 96 × 96 input size DCNN

We studied how the relationship between complexity and classification accuracy by gradually reducing the input size of each image, and changing the number of filters. There was little effect of reducing input size from 256 × 256 pixels to 96 × 96 pixels (classification accuracy dropped from 95.6% to 93.4%, Figure 5a,b), although lower resolution pictures were less accurate. However, this reduces the complexity (and thus processing time) by 10 times, with a relatively small loss of classification accuracy (2.2%). Figure 5c shows the complexity analysis associated with reducing the number of filters for each convolutional layer of input size 96 × 96 AlexNet (AlexNet‐96) (Yousif, Yuan, Kays, & He, 2017b).

By reducing the number of convolutional filters on each layer of AlexNet‐96 DCNN model, we maintained the accuracy over 90% for rapid classification (Table 1). We were able to reduce the classification time from 2,655 ms (accuracy of 95.6%) to 184 ms (accuracy of 93.38%). At this optimization point, we sacrificed 2.22% of the accuracy while the classification time is reduced by 14 times. We compare three image classifiers in terms of accuracy and speed in Table 2. For near real time with reliable performance, we use our DCNN model to classify the image patches.

**Table 1:** Influence of reducing number convolutional layer filters for different convolutional (conv) layers on the accuracy and classification time

conv	# of filters	Accuracy (%)	Time (ms)
1	16	90.06	198
2	32	90.92	138
3	64	93.38	184
4	64	91.24	211
5	32	93.26	252

**Table 2:** Accuracy and classification time per patch among different algorithms classifying images as background, animals, or humans after being trained on 30,000 images

Classifier	Accuracy (%)	Time (ms)
BOW (Fei‐Fei & Perona, 2005; Uijlings, Smeulders, & Scha, 2010)	84.1	786
AlexNet (Krizhevsky et al., 2012)	95.6	2,655
Ours	93.38	184

### 3.2 Object detection evaluation

**Figure 6:**
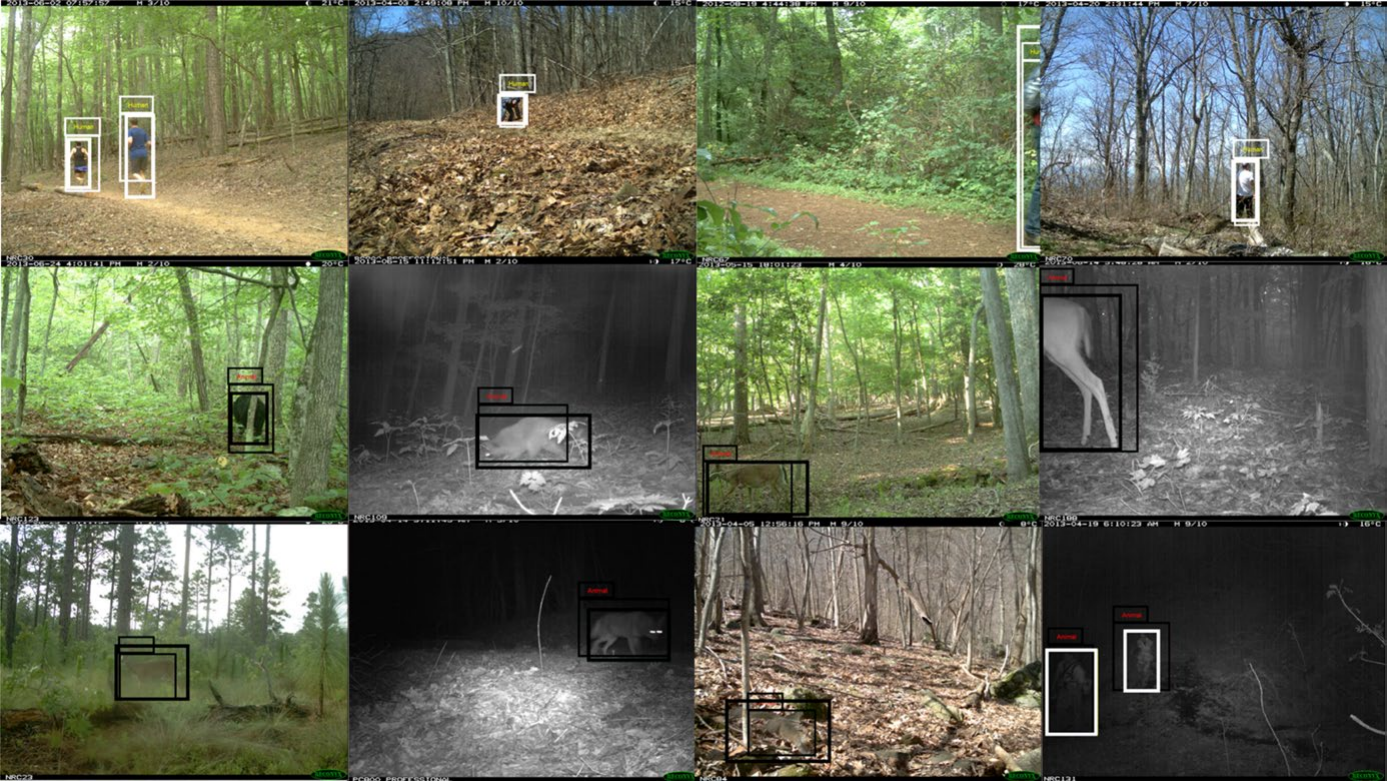
Example detection result of our proposed algorithm classifying humans in white bounding box and animals in black. These show that our method can handle challenging conditions including object deformation, occlusion, and low contrast. A misclassification sample is shown in the last row. Thin and bold boarder bounding boxes refers to our results and ground‐truth, respectively

Our proposed background modeling outperforms other published alternatives in both recall and precision (Table 3), and works even with difficult images typical of camera trapping (Figure 6). In Table 4, we compare our detection results with the other state‐of‐the‐art methods. Again, our method shows a superior result in both performance and time compared with other state‐of‐arts.

**Table 3:** Performance comparison on background subtraction in the Camera Trap dataset with other methods

Method	Recall (%)	Precision
RPCA‐PCP (Candès et al., 2011)	59.56	75.12
ROSL (Shu, Porikli, & Ahuja, 2014)	68.18	80.42
GRASTA (He, Balzano, & Szlam, 2012)	28.84	56.2191
LRGeomCG (Vandereycken, 2013)	58.7	74.59
Deep‐Semi‐NMF (Trigeorgis, Bousmalis, Zafeiriou, & Schuller, 2014)	55.01	72.32
Proposed	73.13	83.55

**Table 4:** Human–animal detection comparison with other methods in our dataset. Metrics showing average detection time per image (seconds) and average human–animal recall

Method	Human–animal recall (%)	CPU time (s) per frame
Faster‐RCNN (Ren et al., 2015)	43.7	19.2
SSD (Liu et al., 2016)	65.2	52.7
RRC (Ren et al., 2017)	33.6	—
Proposed	68.89	0.6

### 3.3 Sequence‐level evaluation

Although our algorithm evaluates individual images, this information can be pooled across sequential frames to classify the contents of a sequence, and then remove the empty sequences and people. We classify a camera trap sequence as (a) background when there is no human/animal is detected, (b) human when all the detected objects are humans, and (c) animal if there is an animal is detected. We evaluated the performance of our detection method based on sequence labeling using six deployments that reflect different camera circumstances that often result in many non‐animal pictures (Table 5).

**Table 5:** Sequence‐level performance evaluation on Camera Trap deployments with different circumstances

Deployment	Description	# of Sequences	# of Images	Animal Recall (%)	FNR (%)	TNR (%)	Accuracy (%)
SD‐1	Mostly Animals	54	835	89.6	100	100	88.7
SD‐2	Animals with false triggers	60	660	100	3.7	5.5	11.7
SD‐3	Mostly Partial animal bodies	73	1,130	88.4	100	100	87.5
SD‐4	Animals with sun spots	64	2,261	98.3	66.67	33.3	90.6
SD‐5	Moving grass	136	4,755	100	0	54.07	54.4
SD‐6	Top‐swaying trees	192	1,395	100	0	30.97	33.9

Note

High values of recall and TNR indicates better object detection and specificity, respectively. While the low values for FNR indicates less misclassifying object as background than misclassifying background as object.

**Figure 7:**
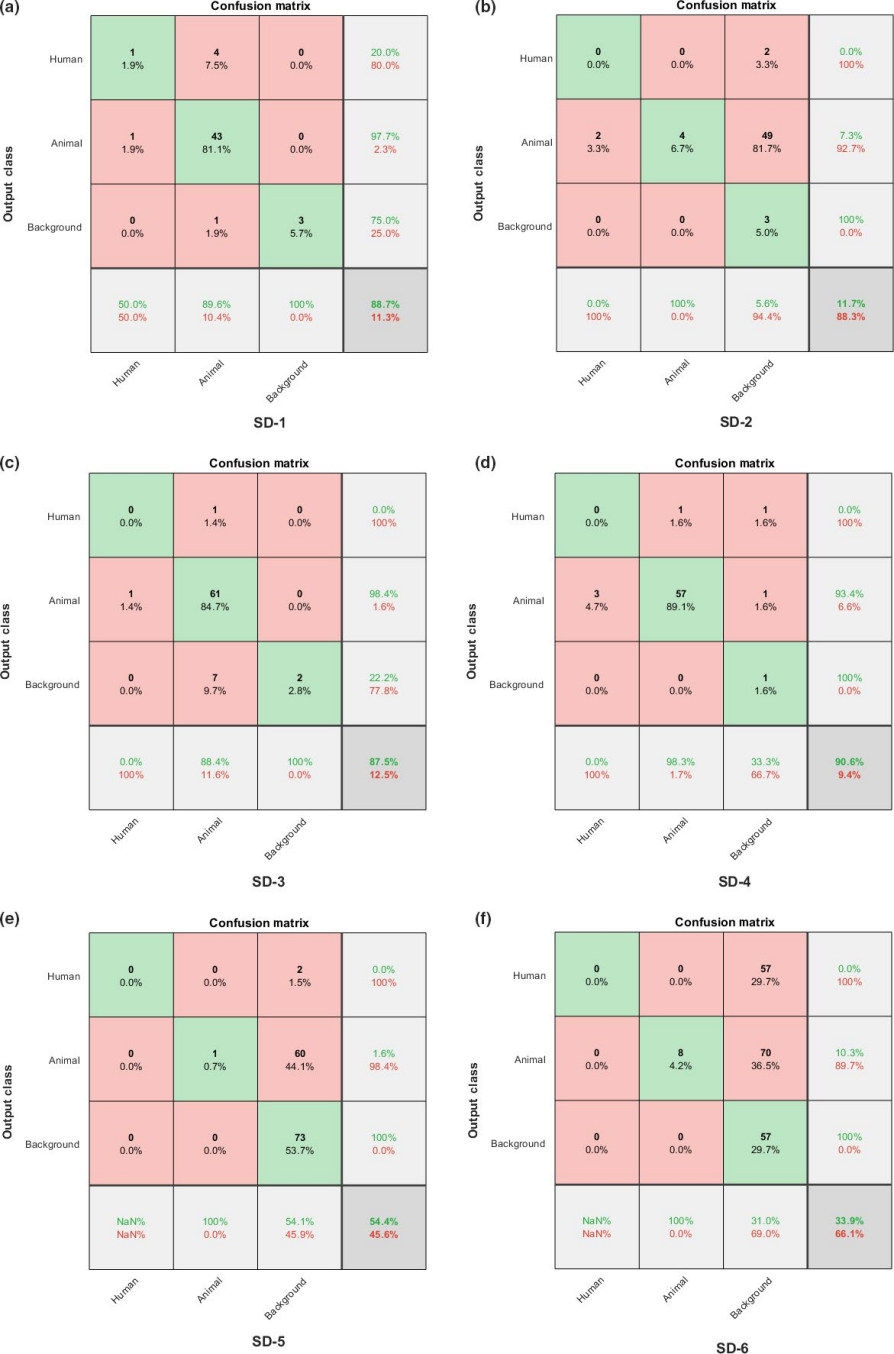
Detailed confusion matrices for six deployments, see Table 5 for descriptions

We evaluate the performance using three different metrics: (a) Recall TP/(TP* *+ FN); (b) False Negative Rate FNR = FN/(FN + FP); (c) True Negative Rate TNR = TN/(TN + FP); and *Accuracy *= (TP + TN)/(TP + TN + FP + FN). For more comprehensive evaluation, we also present detailed confusion matrix results (Table 5; Figure 7). The diagonal cells correspond to correctly classified observations. The off‐diagonal cells correspond to incorrectly classified observations. The column on the far right of the plot shows the percentages precision and false discovery rate. The row at the bottom of the plot shows recall (or true positive rate) and false negative rate. The cell in the bottom right of the plot shows the overall accuracy and error.^4^

**Figure 8:**
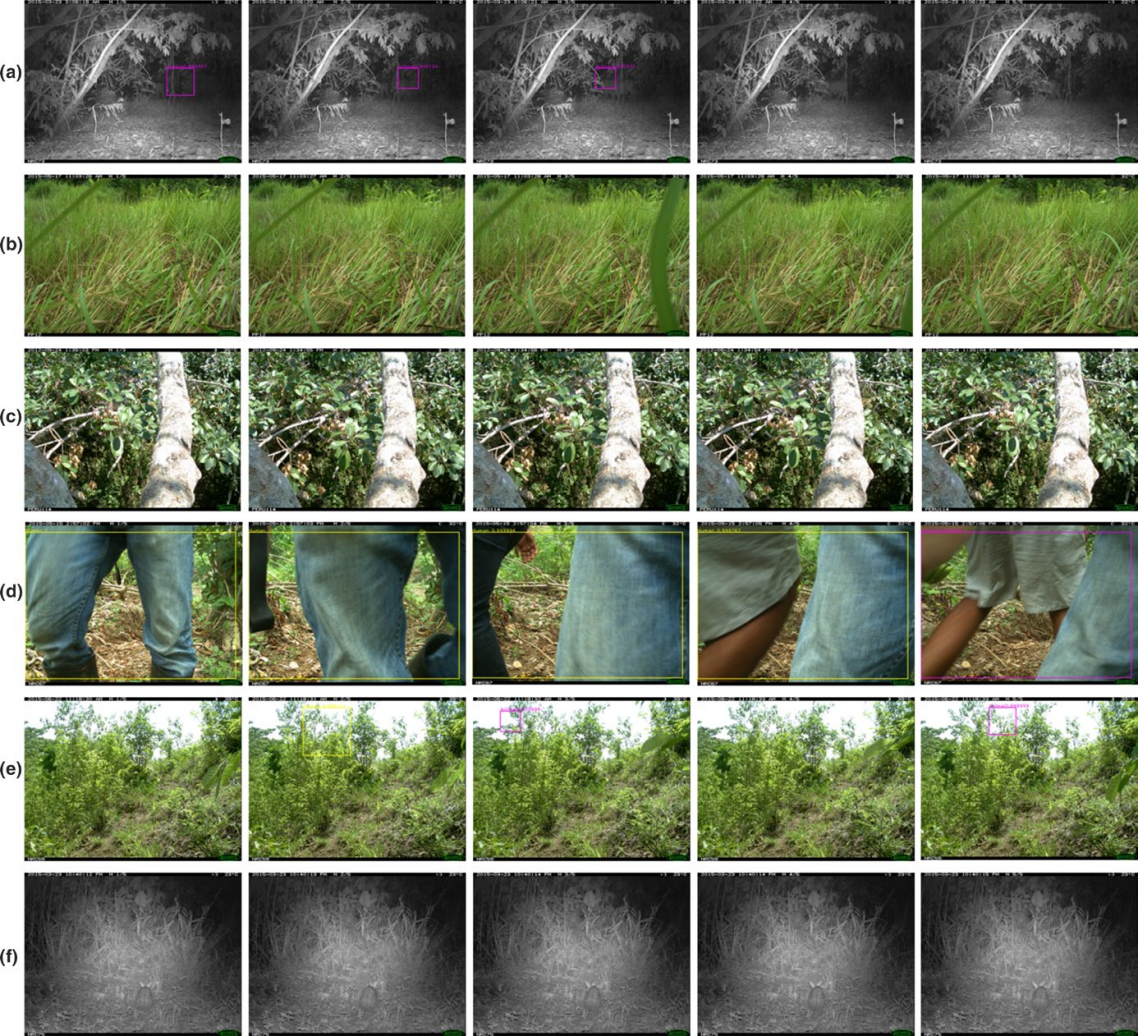
Sample output labeled sequences from each deployment showing three correct identifications (a–c) and three mistakes (d–f). Row a shows the correct identification of an animal (purple bounding box) toward the back of a scene. Rows b and c correctly identify the moving objects as background grass and leaves, respectively, showing no bounding boxes. Row d shows four frames correctly identified as human (yellow bounding box), with the fifth mistakenly classifying the object as an animal. Row e shows moving grass that was classified as an animal and human. Row f shows an animal that was not detected because it did not move during the sequence

In our experiments, the training and testing set are taken from different studies to make sure that the solution can be robust. However, the classifiers are not perfect, and we highlight examples of successful classifications and ongoing challenges in Figure 8.

### 3.4 Image‐level classification evaluation

For this task, we use the camera trap images from Snapshot Serengeti project and have included this with the GUI. The Snapshot Serengeti project is a study with 225 camera traps running continuously in Serengeti National Park, Tanzania, since 2010 (Norouzzadeh et al., 2018; Swanson et al., 2015). For each image, multiple users label the species present, number of individuals, various behaviors, and presence of young. Simple algorithm had been applied to aggregate these individual classifications into a final consensus dataset, yielding a final classification for each image and a measure of agreement among individual answers.

Three main things can be done to deal with data imbalance: ignoring the problem, undersampling the majority classes, or oversampling the minority classes. Because DCNNs need to be learned from vast amounts of data, we choose oversampling. Instead of generating copies from the original training samples, we propose to modify the color contents of the new samples. A large portion of camera trap images are grayscale or have untrue color because of the camera malfunctions. From the experiments, we show that having different color versions from the same scene during training stage leads for better classification. This is mainly caused by making the DCNN use the shape and texture features rather than color features. The first task of analyzing Snapshot Serengeti dataset is to separate the animal frame from the empty frame. We achieve 99.58% in this task while Norouzzadeh *et al.* achieves 96.28%. We choose 80% of images for training and use 20% for testing.

## 4 CONCLUSION AND FUTURE WORK

With the growing reliance of camera traps for wildlife research, there is an increasing interest in developing computer vision tools to overcome the challenges associated with big data projects. Our tool offers an important advance in this effort by helping biologists remove useless images. This is a time‐consuming task, made especially bad in grassy or canopy habitats where 98% or more of the pictures consist only of moving vegetation. The removal of humans is also useful for busy hiking trails where they make up the majority of pictures. Our process to automatically identifying people in pictures could also aid in situations where the privacy of people being photographed is of concern, or in educational programs where school kids are running camera traps and looking through the pictures.

Our model works on both color and infrared photos and has been trained and tested with difficult and challenging images typical of camera trapping. Many camera traps now record video, although this is not often used by biologists because of the added time needed to process. Our algorithms would be more accurate with these higher frame rate sequences, and thus could help biologists bridge the technical gap to make video processing more efficient.

Our algorithms could also be useful for the larger goal of identifying the species of animals in each frame. By recognizing moving objects and placing the bounding boxes around animals, our algorithm prepares camera trap images for automatic identification of species through additional algorithms (He et al., 2016). Using algorithms to identify the species within a bounding box should be much more successful than using the entire frame, for example, when the animal itself is small or only partially in view. Our work also points out the challenge of identifying a non‐moving animals in an image by segmenting each frame into regions using the DCNN feature maps and classify each region with a faster DCNN model (Yousif, Kays, & He, 2017a). The segmented object regions are then verified and fused in the temporal domain using the same background modeling used in this paper that leads to improve the performance by more than 5%. Other object detection methods (i.e., Faster‐RCNN (Ren, He, Girshick, & Sun, 2015), SSD (Liu et al., 2016), and RRC (Ren et al., 2017)) use only the single frame information to find the target object which makes them inefficient for the highly cluttered scene of the camera trap images (Norouzzadeh et al., 2018). Our sequence‐level background subtraction shows an effective approach to localize the moving objects. Most of the recent papers on camera trap images aims to classify the whole image using the available DCNN models. Image‐level classification is unable to (a) localize the object, (b) differentiate between an image that contains a small animal and a background image, and (c) classify a multiple object image.

This work introduces a near real‐time software with outstanding performance compared with the other state‐of‐the‐art. In our unpublished work, we have improved the performance much higher but still can not be run on CPU computers. There is still ongoing work to implement this work beside animal species classification from eastern North America camera trap images as a cloud service.

## CONFLICT OF INTEREST

None declared.

## AUTHOR CONTRIBUTIONS

Hayder Yousif constructed the main ideas of the research, carried out most experiments, and drafted the original manuscript. Jianhe Yuan did the training and testing with DCNN part. Roland Kays and Zhihai He offered useful suggestions for improving the accuracy and revising the manuscript.

### 1 Data Accessibility

The softwares used in this paper have been archived on figshare (https://figshare.com/s/cfc1070ca5a9bdda4cd8).

## ENDNOTES

1
https://drive.google.com/open?xml:id=1c5Hw8TQATdsuTpw_2lyW31RIj5-ck0HM.

2
https://drive.google.com/open?xml:id=1qs_rsypapAqn-UZIqjoINEAXcn875vfY.

3
https://drive.google.com/open?xml:id=1VBwCzg9e0mjMFO-hO_FkLwue2SWzgDzm.

4
https://www.mathworks.com/help.
